# Challenges in laboratory diagnosis and antibiotic treatment options for a newly described Pseudomonas aeruginosa class A beta-lactamase type GES-62 strain

**DOI:** 10.3205/id000099

**Published:** 2025-12-10

**Authors:** Thanh Tuyen Pham, Nils Mungard, Niels Pfennigwerth, Jessica Eisfeld, Sören Gaterman, Sandra Ehrhardt, Milica Lazarevic, Ines Weber, Sylke Lenz-Vogt, Nadine Martzsch, Sebastian Groß, Daniel Ebert, Marta Banach-Schmelzer, Stephan Krieger, Desislava Pantke-Zidarova, Matthias Beese, Askar Yerken, David Avila-Castillo, Dietrich Stoevesandt, Michael Bucher, Matthias Karrasch

**Affiliations:** 1 Department of Laboratory Medicine, Unit III, Clinical Bacteriology Laboratory, Halle University Hospital, Halle (Saale), Germany; 2 Department of Anesthesiology, Halle University Hospital, Halle (Saale), Germany; 3 National Reference Centre for Multidrug-Resistant Gram-Negative Bacteria, Department Medical Microbiology, Ruhr-University Bochum, Germany; 4 Department of Radiology, Halle University Hospital, Halle (Saale), Germany

## Abstract

Antibiotic resistance is a major challenge in modern healthcare, as it severely limits the choice of treatment options. In particular, carbapenemase mediated carbapenem resistance in *Pseudomonas aeruginosa* poses an emerging health risk worldwide. Here, we discovered a hitherto unknown variant of the class A beta-lactamase type GES in a *P. aeruginosa* strain by whole genome sequencing. This multidrug-resistant strain was isolated from bronchoalveolar lavage samples of a 61-year-old man, who suffered from respiratory insufficiency resulting from pneumonia. Ultimately, the patient succumbed to his condition, as there were no further treatment strategies. Given the high drug resistance of *P. aeruginosa* and its increasing role in severe infections, the implementation of methods for the rapid detection of carbapenemases is essential for optimizing therapeutic strategies and preventing nosocomial outbreaks.

## Case description

**Figure 1:**
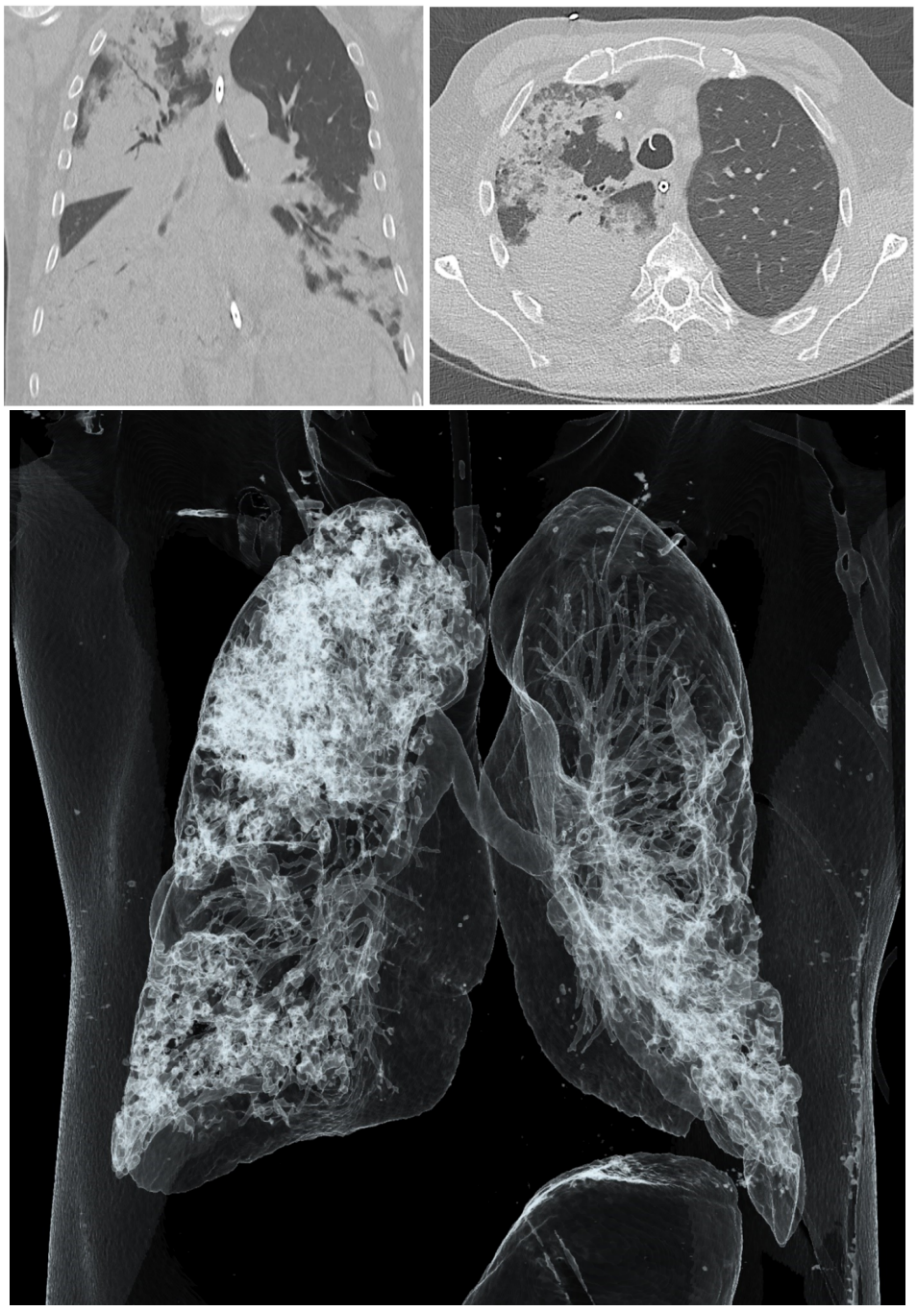
CT imaging of patient’s chest at hospital admission. Upper left panel: Axial non-contrast CT image showing infiltrates in the upper and lower lobes of the right lung. Upper right panel: Coronal non-contrast CT image demonstrating infiltrates in the upper right and both lower lobes of the lung. Lower panel: 3D reconstruction of the CT showing the relative sparing of the left upper lobe.

Carbapenem-mediated resistance, particularly in beta-lactamase-producing *Pseudomonas* *aeruginosa* strains, represents a global threat within hospital settings [1], [2], [3]. A 61-year-old male patient was admitted to our university hospital with respiratory insufficiency resulting from pneumonia. Initially, he was treated with piperacillin-tazobactam. Axial and coronal non-contrast computed tomography (CT) imaging upon admission revealed multiple infiltrates in the right upper lobe and bilateral lower lobes of the lungs (Figure 1 (Fig. 1)). The patient’s anti-infective therapy was adjusted to meropenem, while benzylpenicillin was concurrently prescribed to treat his streptococcal urinary infection. Due to the rapid onset of acute respiratory distress syndrome (ARDS) and sepsis, extracorporeal membrane oxygenation (ECMO) was initiated in our intensive care unit (ICU). During his hospitalization, a multidrug- and carbapenem-resistant *P. aeruginosa* strain was isolated from bronchoalveolar lavage (BAL) samples, prompting an escalation of the antimicrobial therapy to cefiderocol (Table 1 (Tab. 1)). Despite intensive clinical treatment, the patient succumbed to his condition four weeks after admission with persistently elevated infection parameters and progressive clinical deterioration, as there were no further treatment options.

## Methods and results

First, the *P. aeruginosa* strain was isolated from BAL samples and was cultured on blood agar plates for 24 h at 37°C and 5% CO_2_. Pathogen identification was done with mass spectrometry (VITEK MS, bioMérieux, France). Then, antimicrobial susceptibility testing was carried out with an automated microbial testing system (VITEK 2 XL and VITEK 2 Advanced Expert System, bioMérieux, France) and gradient tests (Liofilchem, Italy). The minimum inhibitory concentration (MIC) of antibiotics was interpreted as either susceptible (S), increased exposure (I) or resistant (R) (Table 1 (Tab. 1)), according to EUCAST breakpoints. Thus, the *P. aeruginosa* isolate was classified as a multi-drug resistant gram-negative bacterium and as carbapenem resistant (Table 1 (Tab. 1)).

**Figure 2:**
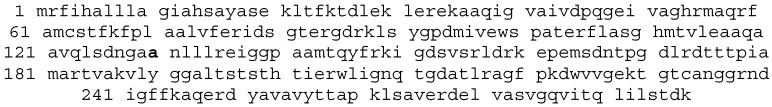
Class A beta-lactamase GES-62 in *Pseudomonas aeruginosa* (strain NRZ-97947) [21]. Class A beta-lactamase GES-62 protein sequence (287 amino acids). GES-62 contains a single amino acid substitution at position 130 compared with the sequence of GES-5 [22], i.e., T130A (marked in bold).

While the carbapenemase rapid test (Coris BioConcept, Belgium; testing for KPC, NDM, VIM, IMP, OXA-48-like) and modified Hodge tests for imipenem, meropenem and ertapenem were negative, the MBL gradient test was positive. This incongruence and high minimum inhibitory concentrationx (MICs) prompted us to further analyze the *P. aeruginosa* isolate on a genotypic level. The multiplex PCR (testing for KPC, IMP, VIM, NDM, OXA-48-like; Xpert Carba-R, Cepheid) and the loop-mediated isothermal amplification (LAMP) system (testing for KPC, NDM, OXA-48-like, VIM, OXA-181-like; eazyplex SuperBug complete C, Amplex Diagnostics), conducted in our clinical laboratory, did not detect any carbapenemases. Concurrently, the strain was subjected to whole-genome sequencing (WGS) at the German National Reference Centre for Multidrug-Resistant Gram-Negative Bacteria (Bochum), where a hitherto unknown variant of the GES-family [4], [5], [6], [7], [8], [9] of carbapenemases was identified (Figure 2 (Fig. 2)). Retrospective testing in our laboratory using an additional carbapenemase LAMP system (testing for IMP, IMI, GES, and GIM; eazyplex SuperBug Expert, AmplexDiagnostics) further confirmed the presence of the GES carbapenemase.

Finally, the new enzyme was designated as class A beta-lactamase GES-62 by NCBI, and was published in the respective database (GenBank: PQ117759.1) [4], [5].

## Discussion and conclusion

Carbapenem resistance in *P. aeruginosa* is multifactorial [10]. Key resistance mechanisms contributing to its nosocomial dissemination include reduced outer membrane permeability, overexpression of drug efflux pumps and the production of inducible beta-lactamases [11], [12], [13], [14], [15], [16]. Moreover, *P. aeruginosa* has the ability to acquire resistance via mutations, further complicating treatment strategies [13]. In this context, the identified class A beta-lactamase GES-62 demonstrated the ability to inactivate carbapenems by hydrolyzing the beta-lactam bond of the antibiotic [17]. Notably, its protein sequence shows high similarity to that of GES-5, a variant known for its clinical relevance and importance [17], [18]. This alignment suggests that GES-62 may exhibit a similar resistance potential. However, detailed analysis of its enzymatic activity is subject for future studies. In contrast, the first identified member of the GES family lacks carbapenemase activity due to its limited carbapenem turnover capacity [17], [18].

In clinical laboratories carbapenemases are detected on a phenotypic (disk diffusion test, MBL E-tests, modified Hodge Test, mass spectrometry) and on a molecular level (PCR, DNA sequencing) [19]. Implementing both simple and sophisticated screening methods can help detect carbapenemases rapidly, which is crucial for the patient’s treatment and for hospital hygiene measures [15]. For *P. aeruginosa* literature [15], [20] recommends to use the inexpensive and simple combined disk method with imipenem and cloxacillin that allows to discriminate between carbapenemase positive and negative strains. Although this screening method was not employed in our laboratory, its implementation is recommended for future diagnostic workflows to quickly screen for carbapenemase producers.

In conclusion, this case highlights the importance and limitations of conventional carbapenemase detection methods, which may fail to identify emerging or uncommon variants. WGS remains the gold standard for comprehensive resistance gene profiling. Early and accurate detection of such carbapenemases is crucial for guiding effective treatment of patients, and preventing and controlling nosocomial infections.

## Notes

### Authors’ contributions

T. T. Pham and N. Mungard contributed equally.

### Competing interests

The authors declare that they have no competing interests.

## Figures and Tables

**Table 1:**
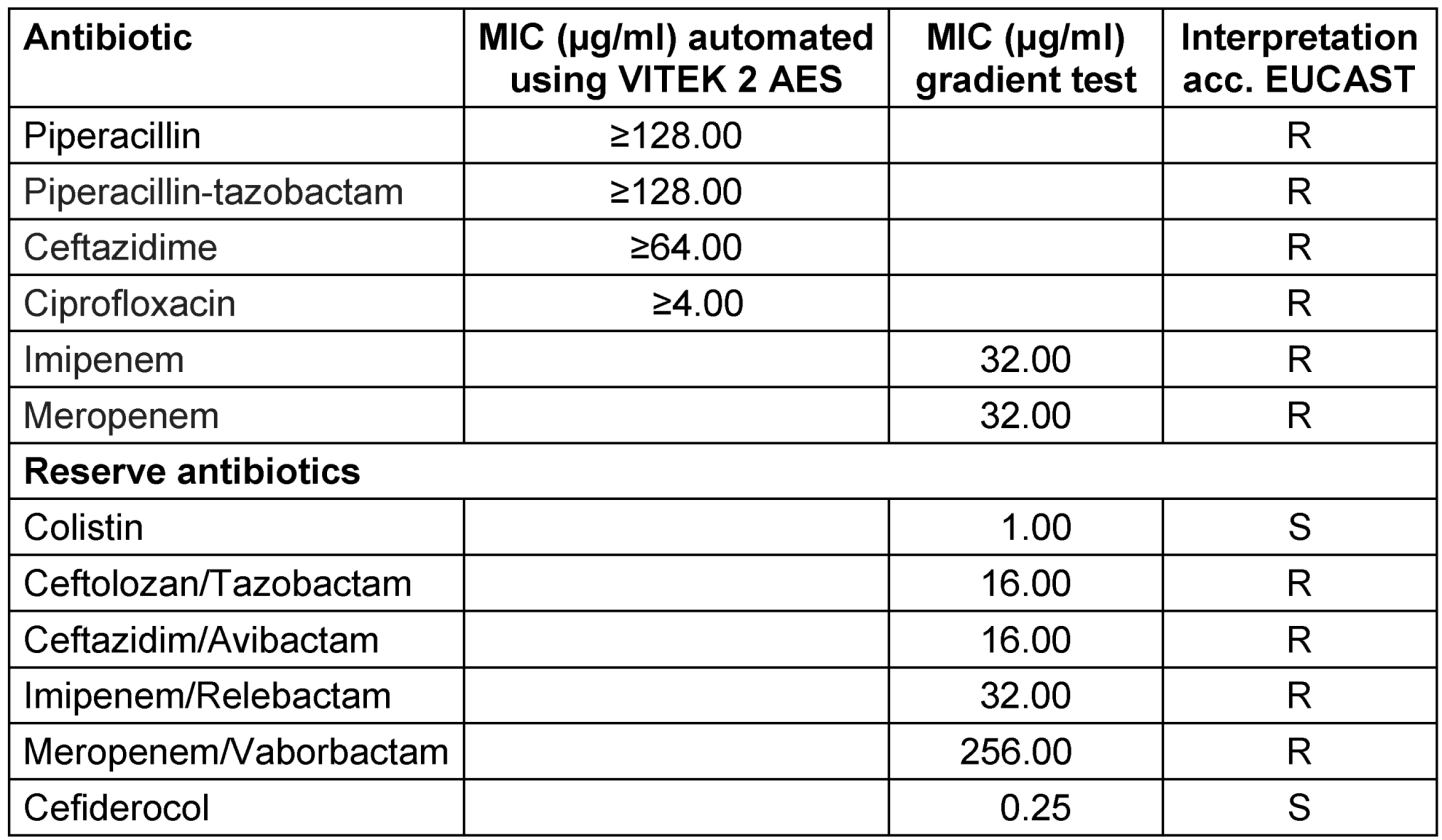
MICs of antibiotics tested against the GES-62 carbapenemase producing *P. aeruginosa* strain. MIC: minimum inhibitory concentration, EUCAST: European Committee on Antimicrobial Susceptibility Testing, S: susceptible, R: resistant
